# Mitochondrial genome characterization of *Gryllodes sigillatus* (Orthoptera: Gryllidae) and its phylogenetic implications

**DOI:** 10.1080/23802359.2021.1899078

**Published:** 2021-03-19

**Authors:** Jianke Yang, Hongliang Dong, Mengqiao He, Jiguang Gao

**Affiliations:** a School of Preclinical Medicine, Wannan Medical College, Wuhu, China; b School of Clinical Medicine, Wannan Medical College, Wuhu, China

**Keywords:** Gryllodes sigillatus, Gryllidae, mitochondrial genome, Phylogeny

## Abstract

*Gryllodes sigillatus* is a cricket widely distributed throughout the world. In this study, we reported the first complete mitogenome sequence of Genus *Gryllodes* and inferred its phylogeny. The mitogenome of *G. sigillatus* was 16,369 bp and consisted of a control region and a typical set of 37 genes. It was AT-rich with strong codon usage bias and possessed a gene arrangement of *trnE-trnS1-trnN*. Phylogenetic analysis indicated *G. sigillatus* was sister species to *Velarifictorus hemelytrus*, together belonging to the Family Gryllidae. Our findings would contribute to understanding mitogenomic evolution and phylogeny of Ensifera.

*Gryllodes sigillatus* (Walker), a field cricket, also known as tropical house cricket, belongs to the Family Gryllidae with a wide distribution throughout the world (Otte 2006). It is a pest but sometimes also kept as a pet or animal feed in addition to a promising protein source for human diet (Ma et al. 2019; Daniso et al. 2020). *G. sigillatus* was first described in 1869 and then classified into different genera many times (Otte 2006). Insect mitogenome sequences have been extensively used to infer the evolution and phylogeny of metazoan at both deep and shallow taxonomic levels due to its fast mutation rate, conservation among conspecifics and lack of recombination (Cameron 2014; Li et al. 2019; Shaoli et al. 2018). Here, we reported the complete mitogenome sequence of *G. sigillatus*, the first in the Genus *Gryllodes*, which would contribute to evolution and phylogeny of Ensifera.

The samples of *G. sigillatus* were collected in the wild grass field from Nankang Town (21°34′41.08″N, 109°24′57.60″E), Beihai, Guangxi Province, China, and stored in the Medical Biology Institute, Wannan Medical College (voucher YGS201001). The species was identified by morphological characteristics and sequence analysis of *cox1* and *cytb*. Genomic DNA was extracted from muscle tissue using a TIANamp Genomic DNA Kit (TIANGEN, Beijing, China) and sequenced by Illumina NovaSeq 6000 platform (Illumina, San Diego, CA, USA). The mitogenome sequence was assembled by GetOrganelle v1.7.0 (Jin et al. 2020), checked manually with Geneious 10.2.2 (Kearse et al. 2012) and annotated using MitoZ version 2.3 (Meng et al. 2019).

The complete mitogenome of *G. sigillatus* was a circular DNA molecule of 16,369 bp in length. It comprised of a control region (also called AT-rich or D-loop region) and a typical set of 37 genes coding for 13 proteins (PCGs), two ribosomal RNA and 22 transfer RNA. Similar to mitogenomes of other Gryllidae species (Ma and Li 2018; Ma et al. 2019), *G. sigillatus* possessed a unique gene arrangement of *trnE-trnS1-trnN*. Twenty genes were encoded on the majority strand (J-strand). The overall AT content was 70.40%. AT- and GC-skew were 0.07 and −0.31, respectively, indicating that the mitogenome of *G. sigillatus* was AT-rich and adenine was preferred. For PCGs, all started with ATN except the *cox1* with TCG as the start codon, and terminated with TAA, TAG or T. The AT percentage of 80.17% at the third codon position was higher than that at other two positions. Moreover, Leu was the most abundant (15.53%) among the used 20 amino acids and codon usage biases were strong with range of relative synonymous codon usage from 0.00 (AGG) to 3.22 (TTA).

To further explore the phylogeny of Grylloidea, we reconstructed the phylogenetic trees based on PCGs sequences of released mitogenomes in GenBank using the maximum likelihood (ML), maximum parsimony (MP) and Bayesian inference (BI) methods that were performed by RaxML GUI 2.0 (Silvestro and Michalak 2012), PAUP* v4.0a168 (Swofford 2003) and MrBayes 3.2.6 (Ronquist et al. 2012), respectively. The substitution saturation was evaluated with DAMBE 7.0 (Xia 2018), and the most suitable models for each of PCGs were assessed by Modeltest-NG v0.1.6 (Darriba et al. 2020).

The topology of ML, MP and BI trees were highly consistent only with slightly difference for support values at some nodes (Figure 1). The results indicated that G. *sigillatus* was sister species to *Velarifictorus hemelytrus* and belonged to Gryllinae subfamily in the Family Gryllidae. *Oecanthus* and *Truljalia* genera grouped together at the base of Gryllidae. It was identical with the result of Ma et al. (Ma et al. 2019). Moreover, the Family Gryllidae, Phalangopsidae and Trigonidiidae clustered a monophyly sharing a gene arrangement of *trnE-trnS1-trnN*, whereas Gryllotalpoidea was at the base of Grylloidea with a *trnN-trnS1-trnE* gene arrangement.

**Figure 1.**
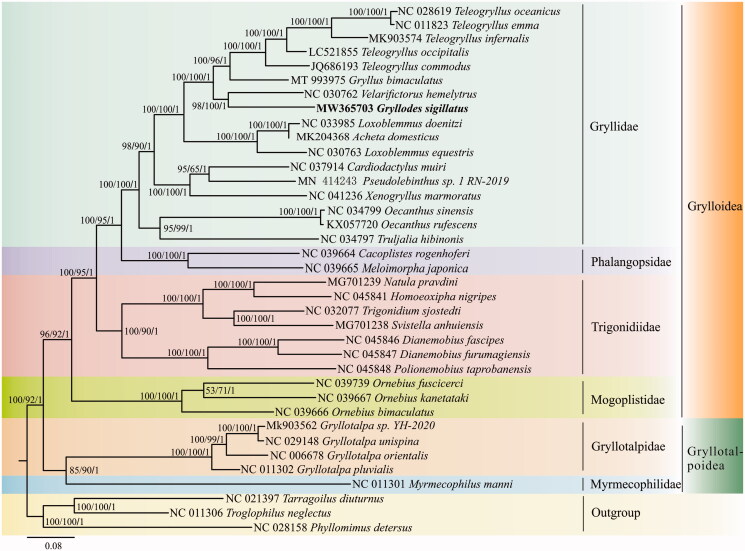
Phylogenetic tree of 37 Ensifera species based on 13 PCGs sequences from mitogenomes and inferred with maximum likelihood (ML), maximum parsimony (MP) and Bayesian inference (BI) methods, respectively. Of them, three mitogenomes NC021397, NC011306 and NC028158 are selected as outgroups. Bootstrap/posterior probability values are displayed on the branches in the order ML/MP/BI, and values less than 50/0.5 are not shown. GenBank accession numbers are listed in front of species name and bold text represents the species in this study.

## Data Availability

The mitogenom sequence data are openly available in GenBank of NCBI at (https://www.ncbi.nlm.nih.gov/) under the accession no. MW365703. The associated BioProject, Bio-Sample, and SRA numbers are PRJNA693646, SAMN17393255, and SRR13495188, respectively.
